# Health is wisely sharing vulnerability

**DOI:** 10.1080/17571472.2016.1193590

**Published:** 2016-06-19

**Authors:** Paul Thomas

Papers in this (8.3) Issue of LJPC continue the theme of improving mental health – this time helping clinicians to be mentally healthy. How can we expect GPs and other primary care practitioners to remain open and empathetic, when so many things threaten our inner balance? How can I expect myself to do this?

The papers are challenging at a very human level. They invite each of us to become more skilled at coming out of our shells and wisely exposing things we prefer to keep hidden – our vulnerabilities. This is not so easy because the fear of being injured when I expose a vulnerability is a real fear. There are plenty of people who are ready to pounce on exposed weakness. And then I have to handle my own self-doubt – anxiety that I might expose the cobwebs in the murkier parts of my mind; or that I might expose to *myself* that I am not my own person, but trapped in a story forced on me by others; or inhabiting a story that I have wilfully adopted without taking responsibility for my actions.

It was Karl Weick who described leadership as ‘the legitimisation of doubt’.[1] It is not knowing everything that marks out a great leader, but knowing when to admit that you don’t know what to do, and then work through this uncertainty to a better place. Often, sharing uncertainty with others helps people to find good collaborative ways forward; and along the way develop trusted relationships that bind them together as a community of friends and followers who will protect each other against those who want to seize the opportunity of someone’s vulnerability to cause them harm. Showing vulnerability may at times be valuable, but it *does* need to be done wisely – in an invited and reciprocal way that harmonises with what others are doing. Making yourself vulnerable can be counter-productive – it makes you open to attack, and simply ‘bleeding all over the place’ is indulgent and portrays you as a victim.

One thing is clear – you can’t understand vulnerability by imagining that people are simple machines. A broken heart cannot be fixed in the way a broken car tyre can be. Changing from being someone who hides their vulnerability to one who values it and uses it wisely is an internal transformation that is inevitably painful – too difficult for many to contemplate. This kind of transformation develops a new sense of who you are – someone who harmonises more than controls.

The idea that harmonising is more important than control for good mental health has been explored in LJPC before. In LJPC 8.2 Hallam presented evidence that music helps people to reach out to others, and in doing this improve their mental health.[2] Her previous research revealed the beneficial effects of music on the child’s growing brain.[3] Her LJPC paper presented new evidence that music helps people over the age of 50 to improve their mental health, including:

• Social benefits – a sense of belonging
• Cognitive benefits – improved concentration and memory
• Health benefits – improved mobility, vitality and feelings of rejuvenation
• Emotional benefits – protection against stress and depression and the development of a sense of purpose

Also in LJPC 8.2 Whitehouse explains why organic metaphors help to understand harmonisation, whereas mechanical metaphors help to understand control. Organic metaphors do this by: ‘*blending ideas from often disconnected corners of our minds … and sharing novel ideas throughout communities of thought*’ … [4]

Learning how to harmonise more than control is helped by organic metaphors and activities that help people to come out of their shells to ‘play’ with others. Engaging in the Arts in small trusted groups and sharing personal stories are powerful ways to do this. However, individuals also have to work alone on their inner selves if they are to modify mechanistic, controlling thinking with harmonising, integrated thinking. No-one can do this for you, as Bob Marley reminds us: ‘*Emancipate yourself from mental slavery* – *none but ourselves can free our minds …*’ This is easier said than done. It means letting go of my need to directly control others, and to try out new rhythms and harmonies that might develop me in unexpected, but satisfying and healthy directions.

In LJPC 8.1, Robert Winston and Rebecca Chicot provided a compelling explanation of what goes on in the brain to sustain harmonising, integrated thinking.[5] They describe the child’s brain as a dynamic, organic system that continually adapts to new experiences by making new synaptic connections – a staggering 700–1000 synapse connections per second! These connections build ways of thinking that shape thinking about everything, including when to harmonise and when to control.

The dynamic nature of the brain is what allows someone to transform their sense of self. They literally ‘rewire’ their brain in response to experience. It is a form of deep learning – Argyris and Schon’s ‘double loop learning’ [6] – that allows internal transformation; a metamorphosis. As Winston points out, such learning has long-lasting effects, and explains why hugs, lullabies and smiles given to a baby help them to achieve later good mental health. More – it alters DNA. The beneficial effects of good parenting (in mice) can be seen two generations later.

In this Issue of LJPC, Luke Kane describes the raw exposure to human suffering that doctors often experience – the type of experiences that cause anxiety and make us want to retract into our shells.[7] In pursuit of better understanding of healthcare systems he worked in an Ebola Treatment Centre in Sierra Leone, so saw at first hand suffering from one of the most serious epidemics of our times. Then he attended the October 2015 WONCA conference in Istanbul, Turkey – where two bombs had just killed hundreds of people.

Thankfully, everyday general practice in the UK lacks such dramatic reminders of the dehumanizing forces in the world. On the other hand, exposure to chronic suffering is an everyday experience for clinicians and it is likely that this lower-intensity but constant exposure will also cause practitioners to retract into their shells to protect their vulnerabilities and anxieties. The UK is experiencing an explosion of anxiety from all manner of insults – social, emotional, physical, financial. GPs are not immune. GPs have anxieties like everyone else – about making mistakes, about hitting targets, about complaints, about the complexity of modern-day general practice, about careers and appraisals and families …

Louise Younie describes how, when leading a medical humanities workshop, she invited participants to describe an image or poem that resonated with them.[8] One senior clinician chose the image of a vulnerable person – because it reminded him of his own vulnerability. This led Younie to realise that being prepared to show vulnerability can be a strength. Clinicians should consider appropriate ways to show their vulnerability in both clinical and leadership roles. She concludes that ‘*the strength of sharing vulnerable spaces may be one of the most generous things we can offer our peers, students or patients*’.

Awareness of the isolation of doctors led Sophie Ratcliffe to set up the Poetry of Medicine project – one-day workshops where literary texts were discussed by participants.[9] The format and the interdisciplinary nature of the workshops allowed participants to articulate issues of their working life that they had previously not noticed or felt unable to speak about. This led her to believe that ‘*enabling this sort of discussion space is critically important to the wellbeing of those who work in the health service*’.

Aditi Sharma and colleagues describe something much more clinical – the important yet-not-so-widely known complication of long-standing Type 1 Diabetes that presents as a palpable breast lump in a young patient.[10] Yet this paper too oozes empathy as the authors consider how to cause her the least suffering when checking that she did not have breast cancer – they conclude that they don’t know.

Younie and Ratcliffe suggest that creating ‘safe spaces’ where health workers can share poetry, images and personal stories with other good and equally imperfect human beings helps to unlock the defences we use to guard or deny our vulnerability. Poetry, images and stories provide a metaphor of reality that is different from the mechanistic metaphor that underpins much thinking in healthcare – a metaphor of harmonisation more than control.

As an adult I doubt that my personal brain is developing anything close to 700 new synaptic connections every second; but I am also fairly sure that a few are still being developed. This suggests that we adults too can emancipate ourselves from mental slavery and transform our inner selves to retract less and reach out more. We can learn how to harmonise rather than control and wisely show vulnerability as we play and sing and laugh and cry with others. And in the process we build our individual and collective senses of self. Co-creative spaces like those of Younie and Ratcliffe sound like a good start.

Paul Thomas Paul.thomas7@nhs.net
